# Effects of Different Parts of the Okra Plant (*Abelmoschus esculentus*) on the Phytosynthesis of Silver Nanoparticles: Evaluation of Synthesis Conditions, Nonlinear Optical and Antibacterial Properties

**DOI:** 10.3390/nano12234174

**Published:** 2022-11-24

**Authors:** G. Roshan Deen, Fatima Al Hannan, Fryad Henari, Sultan Akhtar

**Affiliations:** 1 Materials for Medicine Research Group, School of Medicine, The Royal College of Surgeons in Ireland (RCSI), Medical University of Bahrain, Busaiteen 228, Bahrain; 2 Department of Biophysics, Institute for Research and Medical Consultations (IRMC), Imam Abdulrahman Bin Faisal University, Dammam 31441, Saudi Arabia

**Keywords:** okra leaves, *Abelmoschus esculentus*, silver nanoparticles, surface-plasmon resonance, nonlinear optical property, antibacterial activity

## Abstract

In this work, stable and spherical silver nanoparticles (AgNPs) were synthesized in situ from silver salt (silver nitrate) using the aqueous extract of the okra plant (*Abelmoschus esculentus*) at room temperature and ambient pH conditions. The influences of different parts of the plant (such as the leaves, stems, and pods) on the chemical-reducing effectiveness of silver nitrate to silver nanoparticles were investigated. The aqueous extract of the leaves was found to be more effective in the chemical reduction of silver nanoparticles and in stabilizing them at the same time. The silver nanoparticles produced were stable and did not precipitate even after storage for 1 month. The extract of the stem was less effective in the reduction capacity followed by the extract of the pods. The results indicate that the different amounts of phytochemicals present in the leaves, stems, and pods of the okra plant are responsible for the chemical reduction and stabilizing effect. The silver nanoparticles were characterized by UV-Vis spectroscopy, Fourier-transform infrared spectroscopy (FTIR), transmission electron microscopy (TEM), and energy-dispersive X-ray spectroscopy (EDX). The surface plasmon resonance (SPR) peak at 460 nm confirmed the formation of silver nanoparticles. The nanoparticles were spherical with an average size of 16 nm and polycrystalline with face-centered cubic (fcc) structures. The z-scan technique was used to study the nonlinear refraction and absorption coefficients of AgNPs at wavelengths of 488 and 514 nm under C.W. mode excitation. The nonlinear refraction index and nonlinear absorption coefficients were calculated in the theoretical equations in the experimental data. The antibacterial properties of the nanoparticles were evaluated against Gram-positive and Gram-negative bacteria.

## 1. Introduction

Nanoscience or nanotechnology is a widely used technology in modern translational research. The development of metallic nanoparticles by green chemistry or an eco-friendly approach, using biological materials such as bacteria, fungi, yeast, and plants has gained much research momentum in recent years [1,2,3,4,5]. By this approach, the problems of environmental toxicity and the use of harsh reaction conditions in the synthesis of nanoparticles can be easily circumvented. Among the various biological materials, plants are well suited for the green synthesis of metallic nanoparticles as they are rich sources of phytochemicals, and are non-pathogenic. In addition, the presence of various phytochemicals, such as tannin, flavonoids, proteins, polysaccharides, etc., render the resulting nanoparticles with numerous properties, including antimicrobial, anticancer, and antioxidant activities [6,7,8]. A wide range of metallic and non-metallic nanoparticles, such as silver, gold, copper oxide, zinc oxide, palladium, platinum, titanium dioxide, and iron oxide have been synthesized using the extracts of various plants [6,7,8,9,10,11,12].

*Abelmoschus esculentus*, commonly known as okra, is an important plant with many nutritional and therapeutic values. The plant is a rich source of phytochemicals, such as tannins, alkaloids, carbohydrates, terpenoids, steroids, flavonoids, proteins, and polyphenols. These phytochemicals provide the okra plant with important bioactive antimicrobial, antidiabetic, and antioxidant properties. The aqueous extracts of pulp, flowers, and pods of *A. esculentus* have been used in the synthesis of silver and gold nanoparticles [13]. The different parts of this plant (flowers, pods, stem, and leaves) contain various amounts of phytochemicals, such as polyphenolic compounds, catechins, flavanols, tannins, polysaccharides, glycoproteins, etc. These play a key role in the reduction of metal ions to metal nanoparticles. Gold nanoparticles synthesized using the pulp extract of *A. esculentus* showed good anticancer and antimicrobial properties [14]. Gold nanoparticles synthesized using extracts of immature fruits of *A. esculentus* had strong in vitro antiadhesive activity against Helicobacter pylori [15]. Cadmium oxide and nickel oxide nanoparticles synthesized using the pulp extract of this plant exhibited good antibacterial properties [16].

Among the metallic nanoparticles, silver nanoparticles have received considerable attention owing to their attractive physicochemical and antimicrobial properties [1,4,5,6,7]. These properties allow these nanoparticles to be used in the development of antibacterial surfaces, topical ointments, and wound-healing materials. 

Given the therapeutic values of *Abelmoschus esculentus*, aqueous extracts of different parts of this plant, such as flowers, leaves, pods, and whole plant (as the pulp) have been used in the synthesis of silver nanoparticles [13,14,15,16,17,18,19,20]. A comparative study using different parts of the okra plants on the synthesis of silver nanoparticles has not been conducted, and this forms the subject of this manuscript. 

In this manuscript, we report the synthesis and properties of silver nanoparticles using aqueous extracts of the leaves, stems, and mature pods of *Abelmoschus esculentus.* The effects of the extracts of different parts of the plant on the formation of silver nanoparticles are compared. The characteristics of the nanoparticles were assessed using UV-Vis absorption spectroscopy, electron microscopy (scanning and transmission), energy dispersive X-ray spectroscopy (EDX), and Fourier transform infrared spectroscopy (FTIR). The nonlinear optical properties and the antibacterial properties of AgNPs are also discussed in this manuscript.

## 2. Materials and Methods

### 2.1. Materials

The okra plant was collected from a local garden in Bahrain. The leaves, stems, and leaves were dried in an oven at 60 °C for 24 h. The dried specimens were individually ground to a fine powder in a high-duty blender and stored in airtight glass containers. Silver nitrate (Sigma-Aldrich, MO, USA) was used as received. Milli-Q water collected from the Milli-Q system (Elix Technology, Germany) with a conductivity of 18.2 MΩ cm^−1^ was used for all sample preparations. The antibacterial properties of the synthesized AgNPs were evaluated against three different types of bacteria, *Staphylococcus aureus* (*S. aureus*), *Escherichia coli* (*E. coli*), and *Salmonella typhimurium* (*S. typhimurium*). The bacteria were obtained from the Ministry of Health (MOH, Bahrain; Microbiologics, France).

### 2.2. Preparation of Plant Extract and Synthesis of Silver Nanoparticles

Silver nanoparticles were synthesized using a green method in which individual fresh extracts of the okra leaves, okra stem, and okra seeds were used as the chemical-reducing and -stabilizing agents. The plant extracts prepared were used within 2 h of preparation. The synthesis of silver nanoparticles using a fresh extract of okra leaves is given below as a representative example.

About 2.0 g of okra leaf powder was mixed with 100 mL of water in a 250 mL glass beaker. The mixture was boiled for 20 min under gentle magnetic stirring. The mixture was first filtered using glass wool, and then with Whatman filter paper (No. 1). The extract was pale brown in color and was slightly viscous.

The freshly prepared extract (3 mL) was added to silver nitrate solution (9 mL, 20 mM) in a screw-capped glass vial at 23 °C. The transparent solution immediately turned milky, indicating the rapid formation of silver nanoparticles. The reaction mixture was placed in an orbital shaker for 24 h for the complete reduction of the silver ions to silver nanoparticles. Different concentrations of silver nitrate solution (1, 10, and 20 mM) were used to optimize the maximum yield of silver nanoparticles. The overall optimized reaction condition was observed in 20 mM silver nitrate solution and neutral pH. The silver nanoparticles obtained were purified by repeated centrifugation at 3600 rpm for 15 min followed by washing in water (3 times) to remove water-soluble biomolecules and secondary metabolites. Other samples containing similar concentrations of silver nitrate and okra leaf extract were prepared by heating in a microwave oven for 3, 10, and 20 s. The sample identification codes are the following: S0: no heating, S3: heated for 3 s, S10: heated for 10 s, and S20: heated for 20 s.

### 2.3. UV-Vis Spectroscopy

The formation of silver nanoparticles was studied by measuring the absorbance as a function of time using a double-beam Shimadzu UV-1800 spectrophotometer. The reaction mixture (3 mL) was placed in a quartz cuvette (Hellma Analytics, Germany) of a 1 cm path length, and the absorption spectrum was recorded in the wavelength range 350–700 nm with 1 mm resolution.

### 2.4. Transmission Electron Microscopy (TEM)

The silver nanoparticles were characterized using FEI Morgagni 268 transmission electron microscope (TEM) operating at an accelerating voltage of 80 kV. The samples (S0, S3, S10, and S20) were prepared by depositing a drop of the colloidal nanoparticles on a carbon support copper grid. The grids were air-dried and transferred to the TEM. The electronic images of the silver nanoparticles were obtained in the bright-field imaging mode.

### 2.5. Fourier Transform Infrared (FTIR) Spectroscopy

The infrared spectra of the okra leaves, okra stem, okra seeds, and purified silver nanoparticles, were recorded using a Bruker Alpha spectrophotometer (Bruker Inc., Madison, WI, USA) in the scanning range of 500–4000 cm^−1^. The samples were ground with dry potassium bromide into fine powder using an agate mortar and pestle. The powder was pressed into a thin and transparent pellet using a pellet-making machine.

### 2.6. Energy-Dispersive X-ray Spectroscopy (EDX)

For a wide view of the samples and to determine the purity and chemical compositions of the silver nanoparticles, SEM equipped with an energy-dispersive X-ray (EDX) detector (TESCAN, VEGA 3, 20 kV, Tescan Orsay Holding, Brno, Czech Republic) was carried out on the two selected samples (S0 and S20).

### 2.7. Nonlinear Optical Studies

The nonlinear refractive index and nonlinear absorption of the colloidal silver nanoparticles were measured using the z-scan technique [21]. The technique depends on the intensity variation along the beam waist of the focusing beam and is maximum at the point of focus. The z-scan was performed by translating the sample through the focus of the lens. The transmission from the sample was measured with and without an aperture at the far field of the lens, as the sample was moved through the beam waist using a computer-controlled stepping motor. This allows for the differentiation of the nonlinear refractive index (closed aperture scan) from the nonlinear absorption (open aperture scan). The z-scan was performed using a home-built set-up with an air-cooled multi-line continuous wave (C.W) argon-ion laser at wavelengths of 488 and 514 nm (at 20 mW of power). A lens of focal length 50 mm was used to focus the beam to a spot size of 20 μm with an intensity of 3.2 × 10^7^ W m^−2^.

### 2.8. Antibacterial Activity

The antibacterial activities of the synthesized silver nanoparticles against different types of Gram-positive and Gram-negative bacterial, such as *Staphylococcus aureus* (*S. aureus*), *Escherichia coli* (*E. coli*), and *Salmonella typhimurium* (*S. typhimurium*) were carried out using the Kirby-Bauer disk diffusion susceptibility test method. The bacteria strains were spread on a nutrient agar (LB agar) medium using a sterile spreader in all directions. The disks were loaded with 10 samples (with aseptic precautions) and then the agar plate was incubated at 37 °C for 24 h. The zone of inhibition was observed and measured after 24 h of incubation.

## 3. Results

### 3.1. Formation of Silver Nanoparticles by In Situ Reduction Using Extracts of the Leaves, Stems, and Mature Pods of A. esculentus(okra)

**Figure 1:**
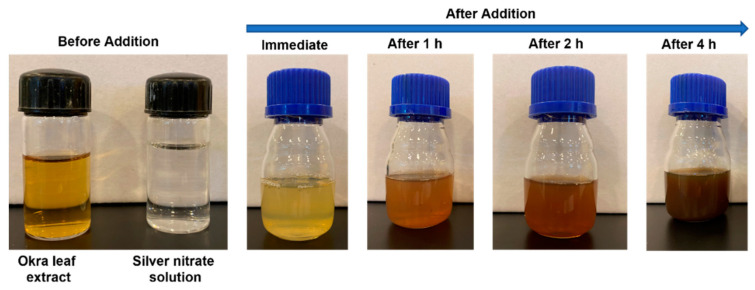
Digital image showing the formation of silver nanoparticles as indicated by color change.

The synthesis of nanoparticles using plant extracts is desirable as the method is environmentally friendly and does not use any toxic chemicals and solvents. The formation of silver nanoparticles using aqueous extracts of different parts of the okra plant, such as leaves, stems, and seeds (pods) was studied using visible observation and UV-Vis absorption spectroscopy. Upon the addition of okra leaf extract to the silver nitrate solution, the clear solution turned opaque and gradually into pale brown indicating the in situ formation of silver nanoparticles. The digital image of the color change as a function of time is shown in Figure 1.

**Figure 2:**
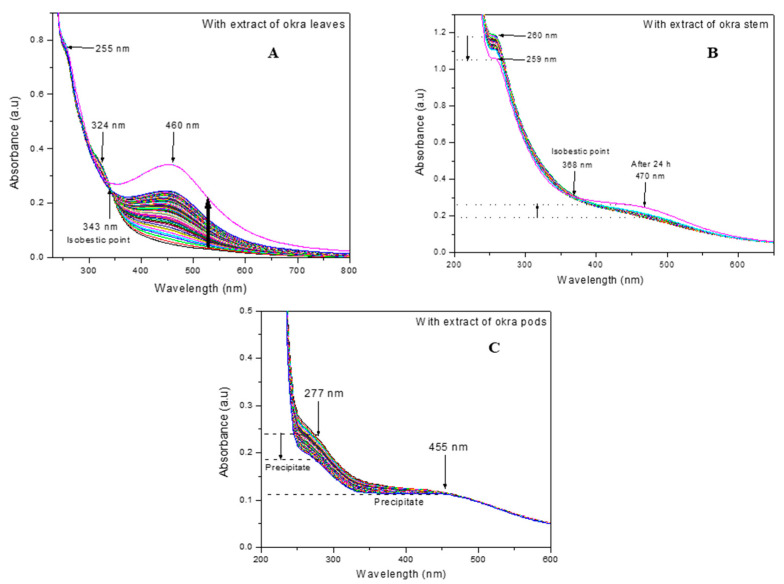
UV-Vis absorption spectrum of silver nanoparticles: (**A**) synthesized using aqueous extract of okra leaves; (**B**) using aqueous extract of okra stem; (**C**) using aqueous extract of the okra pods.

The formation of silver nanoparticles was quantified using UV-Vis absorption spectroscopy. The time-dependent changes in absorption for the formation of silver nanoparticles with extracts of the leaves, stems, and pods are shown in Figure 2A–C.

Interestingly, the formation of silver nanoparticles is more prominent in the sample containing the extract of the leaves (Figure 2A), followed by the sample containing the extract of the stem (Figure 2B).

The absorption spectra of silver nanoparticles prepared with the extract of leaves (Figure 2A) show a gradual increase in absorption with time, indicating the nucleation and formation of the nanoparticles, with a distinct surface plasmon resonance (SPR) peak centered at 460 nm [22]. The gradual increase in absorption intensity at 460 nm clearly shows the gradual reduction of silver ions (Ag^+^) by the phytochemicals of the leaves to silver nanoparticles (Ag^0^). The SPR peak is broad which indicates the nanoparticles are polydisperse. The absorbance peaks at 255 and 324 nm correspond to the conjugated double bonds and the conjugated ring structures of the phytochemicals of the extract of leaves [23]. It can be observed that the absorbance at 324 nm decreases as the absorbance at 460 nm increases with an increase in time. This clearly indicates that the concentration of phytochemicals, which is responsible for the reduction of silver ions, decreases as the concentration of silver nanoparticles increases. In relation to this, an isosbestic point where the absorbance is the same is observed at 343 nm.

The okra leaves are rich in phytochemicals, such as alkaloids, coumarins, flavonoids, glycosides, saponins, steroids, tannins, and terpenoids [18,24]. The rich phytochemicals present in the extract of leaves provide a chemical reduction of silver ions to silver nanoparticles and stabilization of the resulting nanoparticles. The nanoparticles prepared using the extracts of leaves were found to be stable for more than one month, with no obvious change in the SPR peak, indicating that the particles were dispersed in the aqueous solution, with no evidence for aggregation.

The absorption spectra of silver nitrate solution containing the extracts of okra stem and okra pods are shown in Figure 2B,C, respectively. The formation of silver nanoparticles is evident for the sample containing the stem extract with a characteristic SPR peak at 470 nm; however, the magnitude of absorbance intensity is very small, from 0.193 to 0.257. This indicates that the silver nanoparticles formed are low in concentration. The absorbance at 260 nm is attributed to the conjugated double bonds of the phytochemicals present in the stem. The absence of the absorbance peak at 324 nm, which was observed for the extract of leaves, clearly shows that the phytochemical content of the leaves and stem of the okra plant are different.

**Figure 3:**
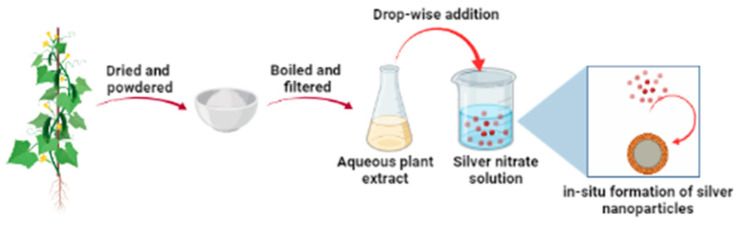
Illustration of the process of in situ synthesis and stabilization of silver nanoparticles.

With the extract of the pods, no formation of silver nanoparticles was observed (Figure 2C) and the SPR peak for silver nanoparticles was absent, instead, a broad shoulder at 455 nm was observed. The broad absorbance peak at 277 nm is attributed to the conjugated double bonds of the phytochemicals present in the pod. With the increase in time, the absorbance intensity of this peak decreased significantly. At the same time, the formation of a fluffy white precipitate was observed in the sample. This is attributed to the complexation and precipitation of silver ions with some phytochemicals present in the okra pods. From this study, it is evident that different parts of the okra plant contain phytochemicals of various concentrations, these play a significant role in the chemical reduction of silver ions and stabilization of silver nanoparticles [18,23,24,25]. The entire process of in situ synthesis and stabilization of silver nanoparticles is illustrated in Figure 3.

### 3.2. Average Number of Silver Atoms per Nanoparticle and Molar Concentration

The average number of silver atoms per nanoparticle (*N_Ave_*) was determined using equation [26],

(1) $$N_{Ave}={\frac{\pi \;\rho \;D}{6\;M}}^{3}\times N_{A}$$

where, *π* = 3.14, *ρ* is the density of the face-centered cubic (fcc) structure (for silver = 10.5 g cm^−3^), *D* is the average diameter of nanoparticles (determined from TEM = 16 nm), *M* is the atomic mass of silver = 107.87 g. *N_A_* is Avogadro’s number = 6.023 × 10^23^ atoms.

Using the above equation and assuming 100% conversion of all silver ions to silver nanoparticles, the average number of silver atoms per silver nanoparticle was determined to be 81 × 10^6^ atoms. The number of surface atoms (*N_s_*) on a single silver nanoparticle was also estimated using the expression, *N_s_ = 4 N_Ave_^*2/3*^*, to be 7.95 × 10^5^ atoms. From the value of *N_Ave_*, the molar mass (*M*_w_) of a single silver nanoparticle was calculated [25] using the relative molar mass of a single silver atom (*M_Ag_*) as, *M_w_* = *M_Ag_* × *N_Ave_* to be 8.74 × 10^9^ g mol^−1^.

The molar concentration of the solution containing silver nanoparticles was calculated using equation [26],

(2) $$C=\frac{N_{Tot}}{N_{Ave}\times V\times N_{A}}$$

where *N_Tot_* is the total number of silver atoms added as AgNO_3_ (0.02 M), *N_Ave_* is the number of silver atoms per nanoparticle (from Equation (1)), *V* is the volume of reaction solution in L (0.026 L), *N_A_* is Avogadro’s number = 6.023 × 10^23^ atoms. The concentration of silver nanoparticles was calculated to be 2.46 × 10^−10^ M.

### 3.3. Functional Group Analysis

**Figure 4:**
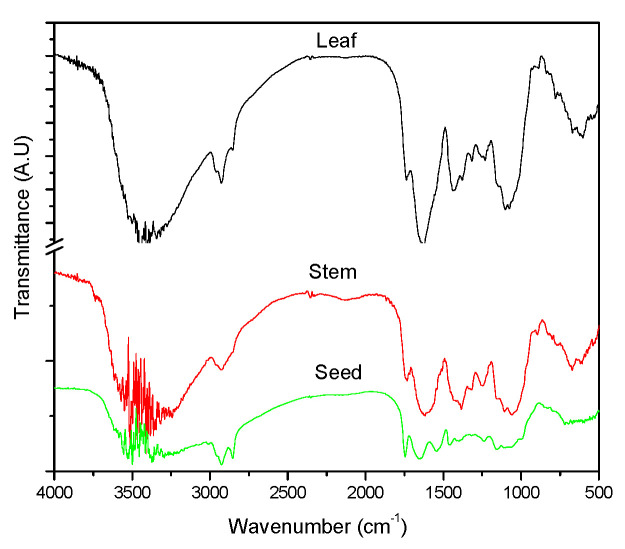
FTIR spectra of the okra leaves, okra stem, and okra seeds.

The major functional groups present in the phytochemicals of the okra leaves, stems, and pods were analyzed using the Fourier transform infrared (FTIR) spectroscopy, and the results are shown in Figure 4. The spectra of the okra leaves, stems, and pods were similar and subtle changes can be identified. The intense broadband with multiple peaks around 3500 cm^−1^ is attributed to the stretching vibrations of hydroxyl groups (OH) [18]. This could arise from the phytochemicals such as glycosides and tannins. The strong absorption peaks at 1632 cm^−1^ (leaf), 1626 cm^−1^ (stem), and 1653 cm^−1^ (pod) correspond to the amide 1 and arise due to the carbonyl stretch vibrations in the amide linkages of proteins. The absorption peak in the range of 1735 cm^−1^ to 1745 cm^−1^ corresponds to carbonyl stretching vibrations of functional groups in aldehydes and carboxylic acids. This peak is sharper and is pronounced for the pods in comparison to the leaves and stem, indicating different chemical compositions of various parts of the okra plant.

### 3.4. Morphology and Size of Silver Nanoparticles

**Figure 5:**
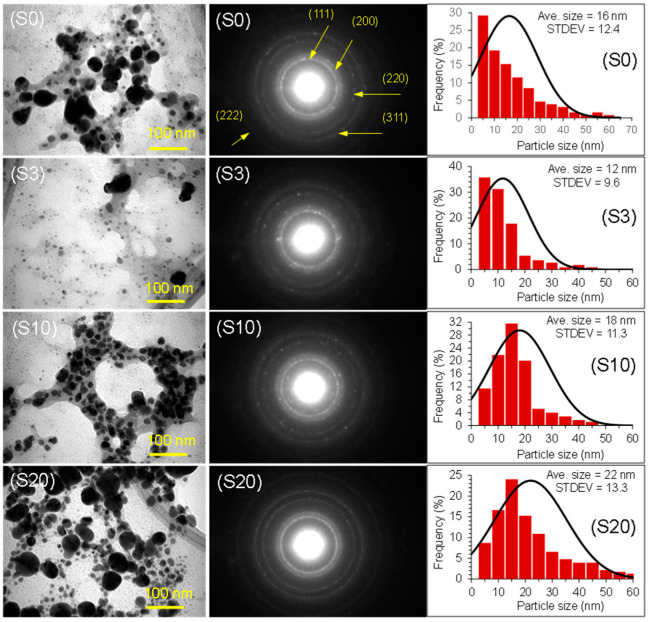
TEM images, SAED pattern, and size distributions of silver nanoparticles.

The surface morphology and size of the silver nanoparticles were studied using transmission electron microscopy (TEM). The transmission electron micrographs, selected area electron diffraction pattern (SAED), and the size histogram of silver nanoparticles prepared using the extract of okra leaves are shown in Figure 5 for all samples (S0, S3, S10, S20). The nanoparticles are predominantly spherical in shape with the following average sizes: S0 = 16 nm, S3: 12 nm, S10: 18 nm, and S20: 22 nm. From the size distribution results, it is apparent that heat treatment did not have any significant effects on the final sizes of the silver nanoparticles. However, only a minor variation in size was observed.

It can be observed that the nanoparticles are capped with thin layers of organic material, which arise from the phytochemicals present in the extract of okra leaves. The SAED pattern is very distinct with Miller indices of 111, 200, 220, 222, and 311, indicating the polycrystalline nature of the silver nanoparticles with face-centered cubic (fcc) structures [27,28]. The results show that the phytochemicals of the okra leaves are highly effective at reducing the silver ions to silver nanoparticles and stabilizing them.

**Figure 6:**
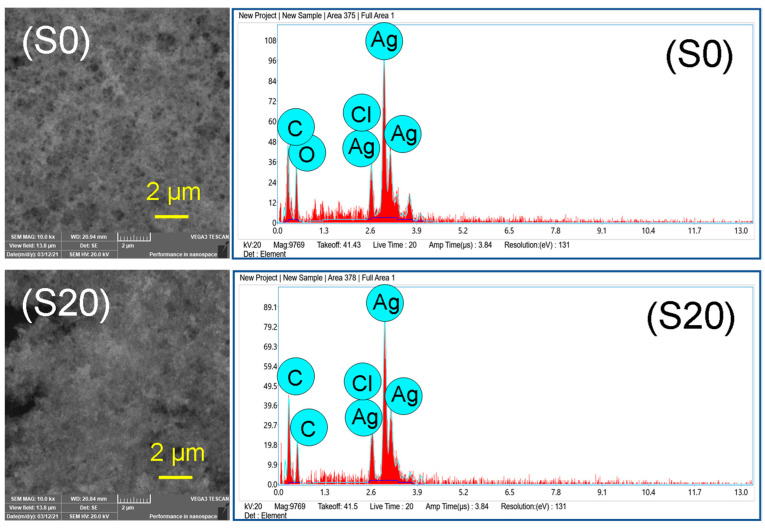
SEM images and EDX spectra of silver nanoparticles, S0 and S20.

The purity and chemical composition of the silver nanoparticles was determined by EDX spectroscopy, and representative spectra of the samples S0 and S20 are shown in Figure 6 along with SEM images. The SEM images revealed well-aggregated silver nanoparticles at a micrometer scale area, indicating the presence of a large number of nanoparticles.

Elemental composition analysis by EDX confirmed the strongest signals for silver atoms (Ag) along with weak signals from carbon (C), oxygen (O), and chlorine (Cl) are observed. Based on the quantitative analysis, the purity of the silver nanoparticles capped with organic material was determined to be 46%. The two other major elements in the sample were carbon and oxygen with compositions of 23% and 21%, respectively.

### 3.5. Nonlinear Optical Property of Silver Nanoparticles

**Figure 7:**
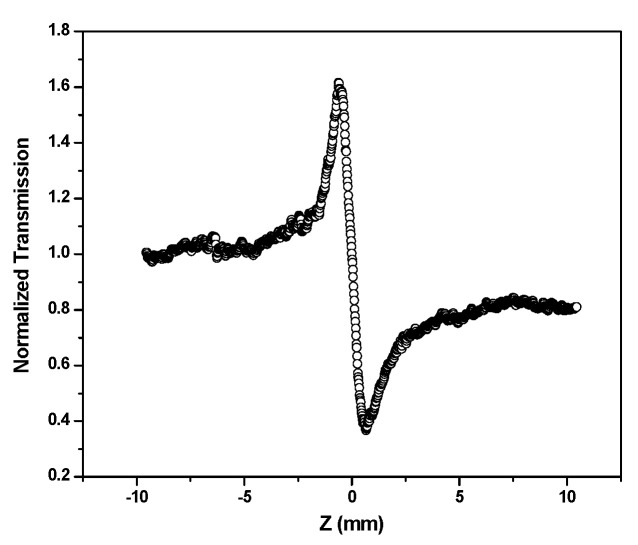
Normalized transmittance (closed aperture) for colloidal AgNPs, S0.

Figure 7 shows the z-scan for the closed case. A typical peak–valley transmission curve is obtained, indicating that the nonlinear refractive index of the medium is negative (self-defocusing).

The nonlinear refractive index arises from the local variation of the refractive index with the temperature due to absorption [29]. The absorption of the laser beam by the sample leads to a spatial variation of temperature in the sample and a consequent spatial variation of the refractive index that acts as the thermal lens resulting in the phase distortion of the beam.

The nonlinear refractive index was calculated from the peak–valley differences of the normalized z-scan curve. The difference between the peak–valley of the normalized transmittance z-scan (Δ*T_p__−v_*) is given by,

(3) $$\Delta T_{p-v}=0.406\;{\left(1-S\right)}^{0.25}\left|\Delta \varphi \right|$$

where |Δ*φ*| is the on-axis nonlinear phase shift at focus and *S* is the linear transmittance of the aperture, which is given by,

(4) $$S=1-e^{-\frac{2r_{a}2}{w_{0}^{2}}}$$

where *r_a_* is the radius of the aperture and *w_*0*_* is the radius of the laser at the entrance of the aperture.

The nonlinear phase shift is given by,

(5) $$n_{2}=\frac{\left|\Delta \varphi \right|\lambda }{2\pi I_{0}L_{eff}}$$

where n_2_ is the nonlinear refractive index, λ is the wavelength of the laser, I_o_ is the intensity at the focus, and *L_eff_* is the effective thickness of the samples. The *L_eff_* is given by,

(6) $$L_{eff}=1-exp^{\frac{-\alpha_{o}I}{\alpha_{o}}}$$

where α_o_ is the linear absorption coefficient, and I_o_ is the intensity at the focus point.

Using the above expressions, the value of the nonlinear refractive index (*n*_2_) at wavelengths 488 and 514 nm was calculated, and the results are shown in Table 1.

**Figure 8:**
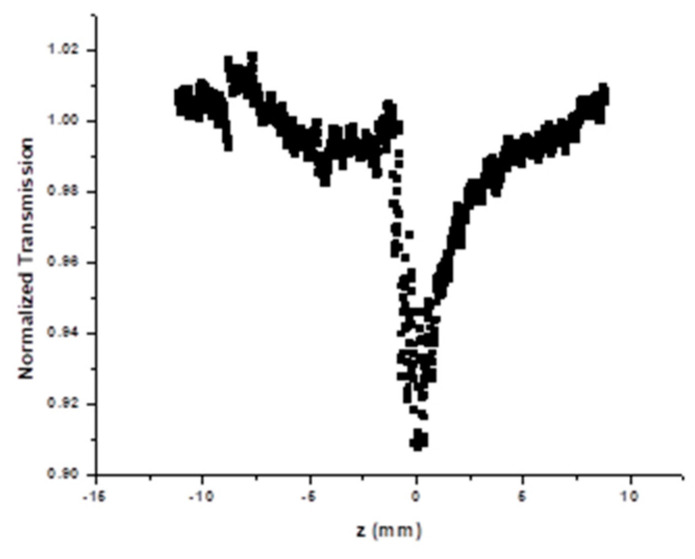
Normalized transmission (open aperture) for colloidal AgNPs, S0 at 514 nm.

The z-scan result of the silver nanoparticles for the open aperture case (normalized transmission) measured at a wavelength of 514 nm is shown in Figure 8.

The transmission peak is symmetric with respect to the focus (z = 0), where the value is minimum. This demonstrates the reverse saturation absorption (RSA) characteristic of silver nanoparticles. The RSA is a positive nonlinear absorption effect, and this feature was also observed at a wavelength of 488 nm.

The normalized transmission for the open z-scan is given by,

(7) $$T=1+\frac{\beta I_{0}L_{eff}}{2\sqrt{2}}$$

where *β* is the nonlinear absorption coefficient at wavelengths of 488 and 514 nm with an intensity of 1.34 × 10^7^ Wm^−2^, given by,

(8) $$\beta =\frac{2\sqrt{2}}{I_{0}\;L_{eff}}$$

The nonlinear absorption coefficient *β* at wavelengths 488 and 514 nm was calculated from the open z-scan, and the results are also shown in Table 1. 

The RSA feature exhibited by the silver nanoparticles can be explained by considering the energy level diagram of noble metals [30,31]. The optical excitations of metals are affected by electron transitions between band *d* and conduction band, *sp*, which occurs due to the energy of photons within the visible region. The excitation wavelengths used in this study for the measurement of nonlinear absorption are 488 nm (E = 2.7 eV) and 514 nm (E = 2.4 eV). The energy values of the two wavelengths are in the vicinity of the SPR peak of silver nanoparticles. The excitation of silver nanoparticles at these two wavelengths involves the transition of electrons in inter-band (*d* to *sp*) and intra-band (*sp* to *sp*). Both these transitions generate free carriers in the conduction band, and these free carriers absorb the photons from the laser beam leading to RSA.

### 3.6. Antibacterial Property of Silver Nanoparticles

**Figure 9:**
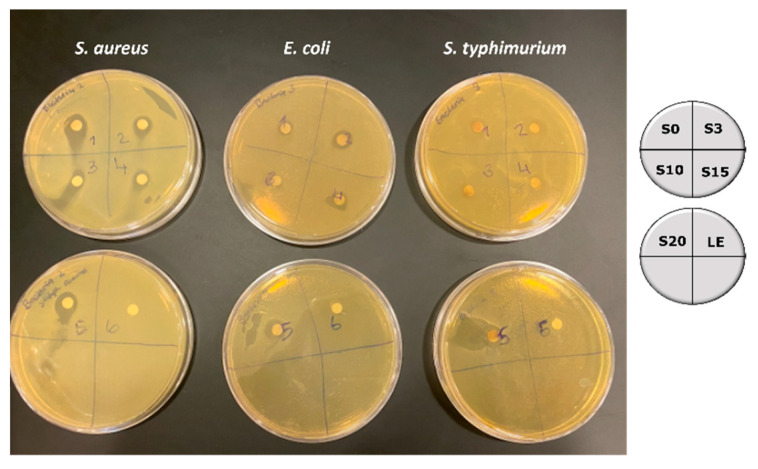
Images of inhibition zones against clinical pathogens by AgNPs.

The antibacterial activity of the synthesized silver nanoparticles was determined against Gram-positive (*Staphylococcus aureus*), and Gram-negative bacteria (*Escherichia coli* and *Salmonella typhimurium*) using a concentration of 20 mM of AgNPs. The diffusion test results on the antibacterial activity of the silver nanoparticles and okra leaf extract as the control (LE) are shown in Figure 9.

For all silver nanoparticles (heated and non-heated during preparation) a clear zone of inhibition in the range of 1 to 1.5 mm is observed. The nanoparticles are more effective against *Staphylococcus aureus* and *Escherichia coli* and least against *Salmonella typhimurium.* The mechanism of interaction is mainly ionic in nature. The ionic interactions between the negatively charged silver nanoparticles and the surface of the bacteria lead to the damage of the cell wall or cell membrane. This damage prevents the synthesis of the new cell wall, making the bacterial cell osmotically fragile [32,33]. It was also observed that all samples had similar antibacterial activity and no direct correlation to their size and morphology was observed. Similar results were reported for silver nanoparticles synthesized using eggshell powder containing collagen as a reducing agent [34].

## 4. Conclusions

Different parts of the okra plant were used to synthesize stable silver nanoparticles. From the results, it is evident that the aqueous extract of the leaves of the okra plant was more effective in the chemical reduction of silver ions to silver nanoparticles and stabilizing them against aggregation, compared to the stem and pods. This difference in chemical activity is attributed to the different levels of phytochemicals present in the leaves, stems, and pods of the okra plant. The initial heating of the sample mixture to speed up the rate of chemical reduction of silver nitrate to silver nanoparticles did not affect the particle morphology and size of the nanoparticles. The silver nanoparticles exhibited nonlinear optical and antibacterial properties. This study has demonstrated the importance of the choice of plant parts and the roles of phytochemical constituents in the synthesis of nanoparticles.

## Figures and Tables

**Table 1:** Absorption (*α*), nonlinear refractive index (*n*_2_), and nonlinear absorption (*β*) values for colloidal silver nanoparticles.

Wavelength, λ (nm)	*α* (cm^−1^)	*n*_2_ (×10^−8^) (cm^2^ W^−1^)	*β* (×10^−6^) (m W^−1^)
488	3.46	3.65	2.80
514	3.18	2.93	2.27

## Data Availability

The (hard and soft copy) data are available via the PI of this research work (RD).
